# Tumor necrosis factor-α-primed mesenchymal stem cell-derived exosomes promote M2 macrophage polarization *via* Galectin-1 and modify intrauterine adhesion on a novel murine model

**DOI:** 10.3389/fimmu.2022.945234

**Published:** 2022-12-16

**Authors:** Jingman Li, Yuchen Pan, Jingjing Yang, Jiali Wang, Qi Jiang, Huan Dou, Yayi Hou

**Affiliations:** ^1^ The State Key Laboratory of Pharmaceutical Biotechnology, Division of Immunology, Medical School, Nanjing University, Nanjing, China; ^2^ Jiangsu International Laboratory of Immunity and Metabolism, The Department of Pathogenic Biology and Immunology, Xuzhou Medical University, Xuzhou, China; ^3^ Jiangsu Key Laboratory of Molecular Medicine, Division of Immunology, Medical School, Nanjing University, Nanjing, China

**Keywords:** intrauterine adhesions, mesenchymal stem cells, exosomes, macrophages, Galectin-1

## Abstract

**Background:**

Intrauterine adhesion (IUA) is a condition caused due to damage or infection of the endometrium. It is characterized by continuous inflammation and following fibrosis and dysfunction. However, the current animal IUA models have several disadvantages, including complex operation, high mortality, and many extra distractions owing to opening of the abdominal cavity to expose the uterus. Mesenchymal stem cells (MSCs), which have been used in treatment of IUA, are heterogeneous and immunosuppressive. However, their therapeutic effect is not as good as expected.

**Methods:**

Here, we successfully built a new murine IUA model, called electric tool-scratching IUA model, and applied it in our experiments to investigate the efficacy of tumor necrosis factor-α (TNF-α) primed MSCs (T-MSCs). In the new model, we used a self-made electric tool that can cause mechanical damage to the endometrium without opening the abdominal cavity. ELISA and histological staining analysis were performed to evaluate pathological features of IUA. qRT-PCR, flow cytometry and immunofluoresence staining were performed to detect the phenotypes of macrophages. TMT proteomics quantification and western blotting assay were performed to analyze the differentially expressed proteins of MSC exosomes.

**Results:**

Based on the new IUA model, we found TNF-α pretreatment could enhance the ability of MSCs to relieve inflammation and reduce endometrium fibrosis. Mechanistically, T-MSC promoted macrophage polarization to M2 phenotype through exosomes. Subsequently, we found the expression of Galectin-1 was increased in T-MSC exosomes. Finally, we analyzed the gene expression pattern of Galectin-1 treated macrophages and found Galectin-1 promoted macrophage polarization to M2 phenotype mainly through the Jak-STAT signaling pathway.

**Conclusions:**

Our studies proposed an innovative mouse model and a better MSC treatment strategy for IUA.

## 1 Introduction

Intrauterine adhesion (IUA), also known as Asherman’s syndrome, refers to damage to the basal layer of the endometrium, resulting in local or complete adhesion of the uterine cavity and cervical canal (1). A small amount of menstruation, recurrent abortion, and amenorrhea are its major clinical manifestations (2). Repeated intrauterine operations, such as dilatation, curettage, and hysteroscopy, are the main causes of IUA (3). IUA is associated with several obstetric complications, including ectopic pregnancy, placental abruption, placenta accreta syndrome, and stillbirth, which seriously harm the reproductive health of women (4). Thus, establishing an appropriate IUA treatment strategy is critical.

IUA is caused by severe endometrial damage or infection, normal structural cell proliferation, and differentiation disorders, fibrous tissue replacement, and adhesion and scar formation (5). Inflammation is an important factor in initiating fibrosis, and continuous inflammation causes fibrosis in local tissues and loss of normal functions (6). Myeloid cells are commonly involved in generating local and systemic immune responses to exogenous or endogenous danger signal–damage-associated molecular patterns and mediating inflammation (7). During the normal menstrual cycle, the endometrium undergoes shedding and regenerative repair, and the subtype of local macrophages changes, suggesting the role of macrophages in endometrium functional repair (8). Following tissue injury, M1 pro-inflammatory macrophages (CD68^+^) clear pathogens while promoting injury, and M2 anti-inflammatory macrophages (CD206^+^) repair and resolve inflammation (9). Thus, promoting the polarization of macrophages to M2 to suppress inflammation quickly might be an effective way to treat IUA.

Mesenchymal stem cells (MSCs) have made excellent progress in the treatment of severe IUA; however, the problem of poor response in some patients still needs to be addressed (10, 11). MSCs are heterogeneous, and they can secrete a variety of substances to regulate immunity, reduce inflammation, and promote tissue repair with changes in the microenvironment (12). We previously reported that pretreatment of MSCs with miRNAs (13, 14), IL-1β (15), or IFN-γ (16) can promote the regulation of macrophages and alleviate inflammatory responses. Further, the anti-inflammatory and repair functions of MSCs depend on their microenvironment and activation state. Studies have also suggested that tumor necrosis factor-α (TNF-α) secreted by inflammatory macrophages can bind to TNFR-1 on bone marrow mesenchymal stem cells (BMSCs) and activate NF-κB/COX2 to upregulate PGE2 for anti-inflammation (17, 18). In recent years, it has also been reported that TNF-α-primed MSCs (T-MSCs) play an important role in retinal nerve cell protection (19), alleviation of transplant immune rejection (20), and other inflammatory diseases (21); however, the role of T-MSCs in IUA treatment is still unknown.

MSC-derived exosomes play an important role in intercellular communication (22). They can promote polarization of macrophages to the M2 anti-inflammatory repair phenotype and play important roles in colitis (23), kidney injury (24), skin wound healing (25), endometrial regeneration (26, 27), and autoimmune diseases (28). Studies have also reported that Galectin-1 ameliorates mouse colitis by conferring oligosaccharide-dependent anti-inflammatory properties on macrophages (29), and the biomaterial containing Galectin-1 inhibits macrophage M1 polarization and promotes M2 polarization, thereby promoting tissue repair (30). Further, glioblastoma cells with defects in the Galectin-1 gene LGALS1 can decrease the percentage of M2 macrophages in the microenvironment (31). Taken together, Galectin-1 can exert anti-inflammatory effects by promoting the polarization of macrophages to the M2 phenotype. We speculated that T-MSC exosomes might contain high Galectin-1 content to exert its anti-inflammatory effect in IUA.

To prove our hypothesis, in this study, we established a murine IUA model to investigate the efficacy of T-MSCs. Notably, we invented a novel murine IUA model and applied it in our experiments. This model will be described in detail later. TNF-α pretreatment enhanced the therapeutic efficacy of MSCs in IUA mice, which was mainly reflected in the inhibition of inflammation. Mechanistically, Galectin-1 in exosomes of T-MSCs promoted macrophage polarization to the M2 phenotype. Thus, our study proposed a novel method to build a murine IUA model and a better MSC treatment strategy for IUA.

## 2 Materials and methods

### 2.1 Animals and experimental protocol

Female Balb/c mice (8–10 weeks old) were purchased from Jiangsu Huachuang Xinnuo Pharmaceutical Technology Co., Ltd. (Taizhou, China), and they were housed in pathogen-free conditions under a 12-h light and dark cycle. All procedures involving mice were approved by the institutional guidelines for animal care and Animal Care Committee at Nanjing University.

The mechanical damage–lipopolysaccharide (LPS) infection (MDLI) IUA mouse model was established using dual methods using uterine curettage and LPS injection as previously described (32). All mice were anesthetized with isoflurane and placed in the supine position. In Result 2, mice were randomly divided into four groups, sham (0 days), in which mice received only an incision wound without any treatment to the endometrium, and three IUA groups (0.5, 3, and 7 days), which received curettage of bilateral intact endometrial with an intrauterine injection of LPS (1 mg/ml). Mice from 0.5-, 3-, and 7-day groups were sacrificed on Days 0.5, 3, and 7, respectively. In Result 3, mice were randomly divided into four groups: sham, IUA, naïve MSCs (N-MSCs), and T-MSCs. MSCs (1 × 10^6^ cells in 200 μl of phosphate-buffered saline (PBS)) were injected through the tail vein 2 h after IUA modeling. Mice from each group were sacrificed on Day 0.5 (early inflammatory phase) and Day 7 (late fibrosis phase).

**Figure 1:**
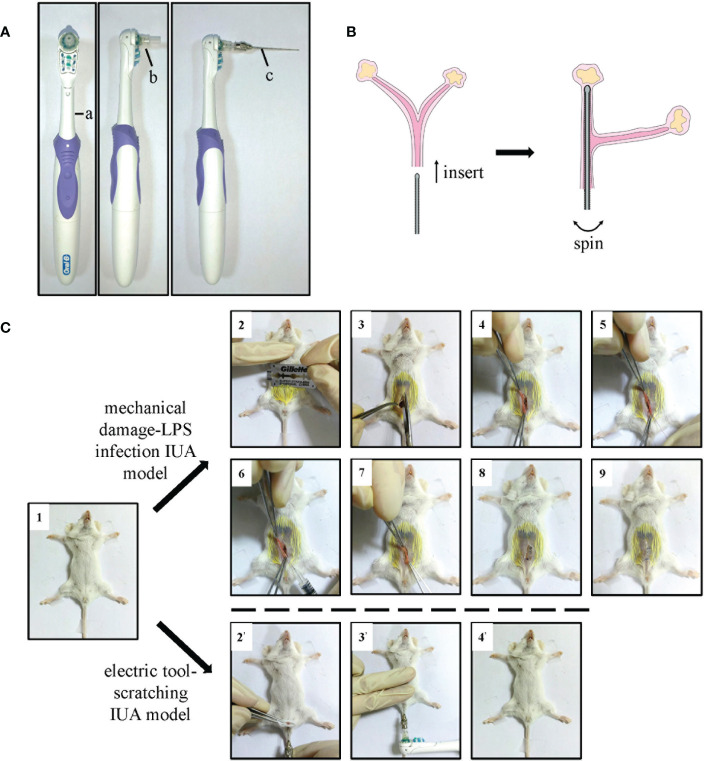
The details of electric tool-scratching IUA model. **(A)** The photos of our electric tool. **(B)** The schematic diagram of modeling process, meaning the needle probe inserted into the uterus of mice. **(C)** The photograph of modeling process. Female Balb/c mice that were 8–10 weeks old were used. IUA, intrauterine adhesion.

The electric tool-scratching IUA mouse model was established as previously described (**Figure 1**). In Result 2, mice were randomly divided into four groups: control (0 days) and three IUA groups (0.5, 3, and 7 days). In the control group, mice received only isoflurane anesthesia. In Result 3, mice were randomly divided into four groups: control, IUA, N-MSCs, and T-MSCs. The MSC treatment and sacrifice approach were the same as above (n = 5–7 in each group). The electric toothbrush used in experiments was purchased from Oral-B (Catalog # 4732).

### 2.2 Cell cultures

All MSCs were human umbilical cord mesenchymal stem cells (hUC-MSCs), and they were purchased from the Nanjing Drum Tower Clinical Stem Cell Center. MSCs were cultured in complete Dulbecco’s modified Eagle’s medium (DMEM)/F12 (Gibco, Grand Island, NY, USA; https://www.thermofisher.com) with 100 μg/ml of penicillin, 10 μg/ml of streptomycin (Invitrogen, Carlsbad, CA, USA; http://www.invitrogen.com), and 10% fetal bovine serum (FBS; Gibco, Australia origin; https://www.thermofisher.com). MSCs between passages 3 and 5 were used for subsequent experiments.

Mouse peritoneal macrophages were obtained from 8- to 10-week-old female Balb/c mice as previously described (33). The mice were allowed to fast for 8 h and sacrificed *via* cervical vertebra dislocation. Following this, 5 ml of cold PBS buffer solution was injected into the abdominal cavity of mice, and they were gently massaged for 2 min. Subsequently, the peritoneal fluid was collected and centrifuged at 300 × *g* for 5 min to obtain cells. If necessary, the erythrocytes were lysed. The peritoneal fluid cells were cultured into cell culture plates with warm DMEM supplemented with 10% FBS for at least 4 h. The supernatant was abandoned, and cells attached to the bottom were washed with warm PBS buffer solution to remove suspension cells. The remaining peritoneal macrophages were used for subsequent experiments.

### 2.3 Chemicals and inhibitors

LPS derived from *Escherichia coli* was purchased from Sigma-Aldrich (St. Louis, MO, USA; Catalog # 0111: B4; https://www.sigmaaldrich.cn). Recombinant mouse TNF-α and human TNF-α derived from *E. coli* were purchased from Novoprotein Scientific Inc. (Summit, NJ, USA; Catalog # CF09 and C008; https://www.novoprotein.com). OTX008 was purchased from MCE (Catalog # HY-19756; https://www.MedChemExpress.cn). Recombinant human Galectin-1 derived from *E. coli* was purchased from R&D Systems (Minneapolis, MN, USA; Catalog # 1152-GA-050; https://www.bio-techne.com). Ruxolitinib (Catalog # SD4740), BAY 11-7082 (Catalog # S1523), and rapamycin (Catalog # S1842) were purchased from Beyotime, Shanghai, China. VX-11e was purchased from ApexBio Technology (Houston, TX, USA; Catalog # A3931, https://www.apexbio.cn/).

### 2.4 Histological and immunofluorescence staining

The mouse uterus samples were fixed with 10% neutral formalin in phosphate buffer. Paraffin-embedded samples were cut into 2-μm sections and then stained with hematoxylin and eosin or Masson’s trichrome stain (Masson). Tissue slices were incubated with primary antibodies against α-smooth muscle actin (α-SMA) (CST, Danvers, MA, USA; Catalog # 19245; https://www.cellsignal.cn/), PCNA (CST, Catalog # 13110; https://www.cellsignal.cn/), and CK19 (CST, Catalog # 12434; https://www.cellsignal.cn/) at 4°C overnight. The sections were exposed to DAB to visualize the antigen signals after incubation with horseradish peroxidase (HRP)-conjugated secondary antibodies, and they were viewed and photographed under a Nikon Eclipse Ti-U microscope equipped with a digital camera (DS-Ri1, Nikon, Tokyo, Japan; https://www.nikoninstruments.com). Tissue slices were incubated with primary antibodies against CD86 (Thermo Fisher Scientific, Waltham, MA, USA; Catalog # 14-0862-82; http://www.thermoscientific.com), CD206 (R&D Systems, Catalog # AF2535; https://www.bio-techne.com), and F4/80 (Abcam, Cambridge, UK; Catalog # ab6640; https://www.abcam.cn/) at 4°C overnight. After being rinsed in PBS three times, the samples were incubated with Alexa Fluor 488- and 647-conjugated secondary antibodies at room temperature in the dark for 1.5 h, and nuclei were then stained with DAPI (Bioword, Nanjing, China). Slides were visualized using an FV3000 Laser Scanning Confocal Microscope (Olympus, Tokyo, Japan; https://www.olympus-global.com/). Staining was quantified from five random fields per sample with a mean value using ImageJ.

### 2.5 Cells treatment and co-culture

MSCs were stimulated with TNF-α (10 ng/ml) for 24 h to prepare T-MSCs. OTX008 (60 μM) was added into MSCs 1 h before TNF-α addition to inhibit Galectin-1 expression. MSCs or carboxyfluorescein succinimidyl ester (CFSE)-marked MSCs were added into the mouse peritoneal macrophage culture environment (1:10) directly or with transwell for 24 h. MSCs were sustained with CFSE (2.5 μg/1 × 10^7^ cells) purchased from Invitrogen (Thermo Fisher Scientific, Inc., Waltham, MA, USA) for 10 min to obtain CFSE-marked MSCs. MSC-derived exosomes (25 μg/ml) were added into the macrophage culture environment for 24 h. Recombinant human Galectin-1 (1 μg/ml) was used to treat mouse peritoneal macrophages for 24 h. Ruxolitinib (1 μmol/L), BAY 11-7082 (1 μmol/L), VX-11e (100 nmol/L), and rapamycin (10 μmol/L) were used to pretreat mouse peritoneal macrophages for 1 h.

### 2.6 Quantitative real-time PCR

Total RNA was extracted from tissues, cells, or exosomes using TRIzol reagent (Vazyme Biotech Co., Ltd., Nanjing, China; https://www.vazyme.com/) following the manufacturer’s instructions. The concentration and purity of RNA were determined by measuring the absorbance at 260 and 280 nm using NanoDrop 2000. Total RNA (1 μg) was reverse transcribed in a 20 μl system using HiScript ll Q RT SuperMix for qPCR (Vazyme Biotech Co., Ltd., Catalog # R222-01; https://www.vazyme.com/). Subsequently, quantitative real-time PCR was performed using the SYBRGreen PCR Master Mix (with Rox) (Invitrogen) and Step-one plus Real-Time PCR System (Applied Biosystems, Foster City, CA, USA; https://www.thermofisher.com). The primer sequences are shown in **Supplementary Table 1**.

### 2.7 Enzyme-linked immunosorbent assay

Protein levels of IL-1β, IL-6, and TNF-α in mouse serum were detected using the corresponding mouse ELISA kit according to the manufacturer’s instructions (BioLegend, San Diego, CA, USA; http://www.biolegend.com/). The protein level of Galectin-1 in the cell culture supernatants was detected using Human Galectin-1 Quantikine ELISA kit (R&D Systems, Catalog # DGAL10; https://www.bio-techne.com).

### 2.8 Flow cytometry assay

PE anti-human CD34 (Catalog # 12-0349-41), CD45 (Catalog # 12-0459-41), HLA-DR (Catalog # 12-9956-41), CD90 (Catalog # 12-0909-42), CD73 (Catalog # 12-0739-42), CD105 (Catalog # 12-1057-41), and mouse IgG1 kappa Isotype Control (Catalog # 12-4714-81; Thermo Fisher Scientific, Rockford, IL, USA; http://www.thermoscientific.com) were used for labeling hUC-MSCs. PE anti-mouse CD206 (Catalog # 141706) and APC anti-mouse F4/80 (Catalog # 157306; BioLegend, Enabling Legendary Discovery, San Diego, CA, USA) were used for labeling macrophages. Annexin V-FITC/PI Apoptosis Detection Kit (Catalog # A211-01; Vazyme Biotech Co., Ltd. (https://www.vazyme.com/)) was used for the detection of apoptosis in cells. All flow cytometry data were acquired using the BD FACSCalibur cytometer (BD Biosciences, San Diego, CA, USA; http://www.bdbiosciences.com) and analyzed using FlowJo software (Treestar, Inc., San Carlos, CA, USA; http://www.treestar.com).

### 2.9 Western blotting

Western blotting was performed as previously described (34). Protein concentrations were determined using the Bradford assay (Pierce, Thermo Scientific, Rockford, IL, USA; http://www.thermoscientific.com). The total protein content (30 μg) of each sample was subjected to sodium dodecyl sulfate–polyacrylamide gel electrophoresis (SDS-PAGE) and immunoblotted with the desired antibodies against CD63 (Catalog # sc-15363, Santa Cruz, Dallas, TX, USA; http://www.scbt.com), Galectin-1 (Catalog # MA5-32779, Invitrogen; https://www.thermofisher.cn), and GAPDH (Catalog # AP0063, Bioworld; https://www.bioworld.com).

### 2.10 Cell viability assay

To assess the effect of TNF-α on cell viability, a Cell Counting Kit-8 **(**CCK-8**)** assay was used according to the manufacturer’s instructions (Catalog # CK04, Dojindo, Tokyo, Japan). hUC-MSCs (P5) were seeded onto 96-well plates at a concentration of ∼5 × 10^3^ cells/well. Different concentrations of TNF-α were used to treat cells for different time duration**s** as described in the article.

### 2.11 Exosome isolation and characterization

N-MSCs or T-MSCs were cultured in DMED/F12 containing exosome-depleted FBS (by 18-h centrifugation at 100,000 *g*) for 24 h. Exosomes from culture supernatants were isolated using VEX Exosome Isolation Reagent (Catalog # R601, Vazyme Biotech Co., Ltd.; https://www.vazyme.com/) according to the manufacturer’s instructions. The pellet was resuspended in PBS and sterilized *via* filtration through a 0.22-μm filter (Millipore, Darmstadt, Germany; http://www.emdmillipore.com). In each exosome preparation, the concentration of total proteins was quantified using a Bradford assay (Pierce) and then stored at −80°C until further use. Protein markers of purified exosomes were determined using Western blotting with the anti-CD63 mentioned above. The size distribution of particles and characteristic markers CD63 and CD9 were measured using Nanoflowmeter (NanoFCM Inc., Xiamen, China; http://www.nanofcm.com). The transmission electron micrographs of exosomes were carried out in Shanghai XP Biomed Ltd. (http://www.xpbiomed.com/).

### 2.12 Screening of differentially expressed proteins and signaling pathway

Exosome samples were analyzed using TMT proteomics quantification, and mouse peritoneal macrophage samples were analyzed *via* RNA sequencing in Hangzhou Lianchuan Biotechnology Co., Ltd. (Hangzhou, China). Preliminary progression was analyzed after obtaining raw data. The fold value represented the degree of differential expression between the experimental and control groups. The standard used to judge differential expression was as follows: the protein or gene expression from control groups was used as a valid value. Compared to that of the experimental groups, a fold change of <1.0 indicated a downregulated protein/gene, while a fold change of >1.0 indicated an upregulated protein/gene. Genes with a fold change of >1.5 or <0.5 compared to those in the control group were selected for further analysis.

### 2.13 Statistical analysis

All values presented on the graphs are shown as means ± SEM. ANOVA and unpaired Student’s t-tests were used to analyze statistical significance, and p-values < 0.05 were considered statistically significant.

## 3 Results

### 3.1 A novel way to build murine intrauterine adhesion model–the electric tool-scratching model

Appropriate animal models are critical for studying the mechanism, prevention, and treatment of disease. There are many kinds of IUA models, and the most used is the MDLI IUA model. However, existing models have many shortcomings, including highly traumatic surgery, high infection and death rates, and complex and time-consuming operation. The details will be explained in the Discussion section.

Therefore, a better IUA animal model is urgently needed. We invented a new tool that can cause mechanical damage to the uterus without other wounds. A utility model patent (Patent No. ZL 2020 2 1895669.9) has been applied for this tool. It consists of a shaking handle containing an electric motor (**Figure 1A** a), a needle probe with a rough surface (**Figure 1A** c), and a connector to connect the probe to the handle (**Figure 1A** b). We removed the bristles of an electric toothbrush and used it as the shaking handle. We then stuck the head of a 1-ml syringe to act as the connector. We painted a thin layer of epoxy resin adhesive onto a lavage needle (#6) and then scratched it with a scissor to make a rough surface. The size of the uterus of 8–10 weeks’ old female Balb/c mice was approximately 27 mm in length and 0.7 mm in diameter, which is just enough to fill up with a #6 gavage needle. When the probe was inserted into the uterus of mice and the switch on the handle was pressed, it intensively shook and caused mechanical damage to the endometrium (**Figure 1B**).

We could clearly notice the advantages of the new IUA model by comparing its modeling processes with those of the MDLI-IUA model. The process was as follows: 1) female mice were anesthetized with isoflurane and fixed in the supine position; 2) they were then disinfected, and hair from their abdominal surfaces was removed; 3) the abdominal skin and muscles were then cut; 4) one side of the uterine horn was found; 5) a syringe needle was inserted, and the lining of their uterus was scratched 50 times; 6) 10 µl of LPS–saline solution was then injected into their uterine cavity; 7) both ends of the uterine horn were held with forceps for 5 min to ensure that LPS is fully absorbed by their tissues; 8) their muscle layer and skin were sutured; 9) the mice were put under a warm lamp for recovery from anesthesia. By contrast, the operation of the new method was simpler: 1) female mice were anesthetized with isoflurane and fixed in the supine position; 2) the labia of mice were gently lifted with forceps, and the probe of the IUA modeling tool was inserted into the uterus of mice through the vaginal opening; 3) the switch was pressed, shaken for 8 s, and then paused for 8 s. This process was repeated twice more; 4) the probe was slowly pulled out, and the mouse was placed under a warm lamp for recovery from anesthesia (**Figure 1C**). In this way, a new murine IUA model, called the electric tool-scratching IUA model, was built.

### 3.2 Electric tool-scratching intrauterine adhesion model can achieve nearly identical effects as those of mechanical damage–lipopolysaccharide infection–intrauterine adhesion model

**Figure 2:**
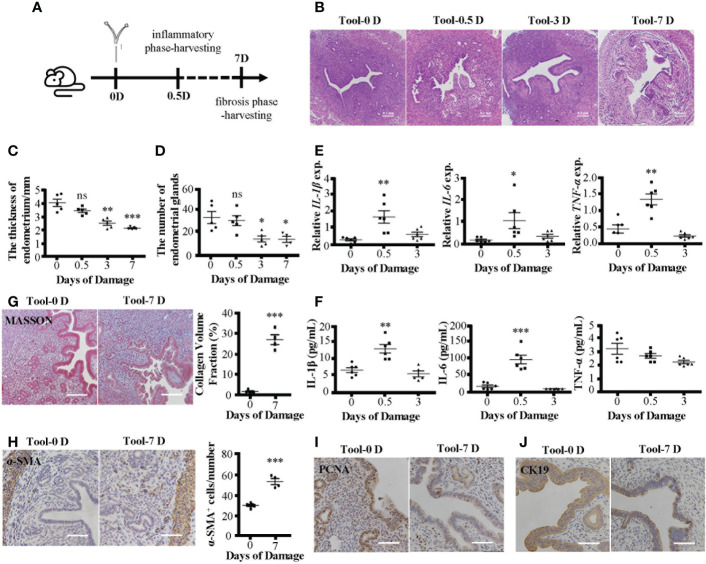
Pathological features of electric tool-scratching IUA mice model. **(A)** The schematic diagram of modeling process. IUA mice model was established by electric tool-scratching way mentioned before. The early inflammatory phase and late fibrosis period respectively are 0.5 and 7 days after modeling. **(B)** H&E staining of murine uterine tissues. **(C)** The statistical figure of the endometrial thickness. **(D)** The statistical figure of gland numbers. **(E)** Relative expression of mRNAs of IL-1β, IL-6, and TNF-α in murine uterine tissues detected by qRT-PCR assay. **(F)** The level of IL-1β, IL-6, and TNF-α in serum detected by ELISA. **(G)** Masson’s trichrome staining of murine uterine tissues and statistical figure of collagen volume fraction. **(H)** The α-SMA protein expression detected by immunohistochemistry and statistical figure of numbers of α-SMA^+^ cells. **(I)** The PCNA protein expression detected by immunohistochemistry. **(J)** The CK19 protein expression detected by immunohistochemistry. Bar = 0.1 mm. The measurement data are presented as the means ± SEM, n = 6; *p < 0.05, **p < 0.01, ***p < 0.001. IUA, intrauterine adhesion; α-SMA, α-smooth muscle actin. ns, no significance.

Consistent with the MDLI-IUA model, the new IUA model showed pathological features of IUA. We prepared two kinds of IUA models (**Figure 2A**; **Supplementary Figure 1A**), and both showed damaged endometrial structure (**Figure 2B**; **Supplementary Figure 1B**), thinning endometrial thickness (**Figure 2C**; **Supplementary Figure 1C**), and decreasing number of endometrial glands as time goes on (**Figure 2D**; **Supplementary Figure 1D**). In the early stages (D0.5), the mRNA expression levels of inflammatory cytokines (IL-1β, IL-6, and TNF-α) in uterine tissues increased in the IUA mice (**Figure 2E**; **Supplementary Figure 1E**). Meanwhile, their serum levels increased in the MDLI-IUA model (**Supplementary Figure 1F**). However, serum expression levels of IL-1β and IL-6 but not TNF-α increased in electric tool-scratching IUA mice (**Figure 2F**). In the fibrosis phase (D7), collagen deposition became serious (**Figure 2G**; **Supplementary Figure 1G**), and α-SMA protein levels in uterine tissues significantly increased (**Figure 2H**; **Supplementary Figure 1H**) in the IUA mice. The expression of PCNA (**Figure 2I**, **Supplementary Figure 1I**) and CK19 (**Figure 2I**, **Supplementary Figure 1I**) decreased in uterine tissues of IUA mice, representing the proliferation level of endometrium and number of epithelial cells, respectively. According to these data, the electric tool-scratching IUA model could achieve nearly identical effects as those of the MDLI-IUA model.

### 3.3 TNF-α pretreatment enhances the therapeutic efficacy of mesenchymal stem cells in intrauterine adhesion mice

To improve the efficacy of MSC-based therapies for IUA, we used the TNF-α to pretreat MSCs. First, the T-MSCs retained their surface markers, including the negative expression of CD34, CD45, and HLA-DR and the positive expression of CD73, CD90, and CD105 (**Supplementary Figure 2A**). Second, TNF-α pretreatment did not alter cell viability (**Supplementary Figure 2B**) or induce cell apoptosis (**Supplementary Figure 2C**). However, the mRNA (**Supplementary Figure 2D**) and protein (**Supplementary Figure 2E**) expression levels of TRAF-1 (the receptor of TNF-α) were upregulated in T-MSCs.

**Figure 3:**
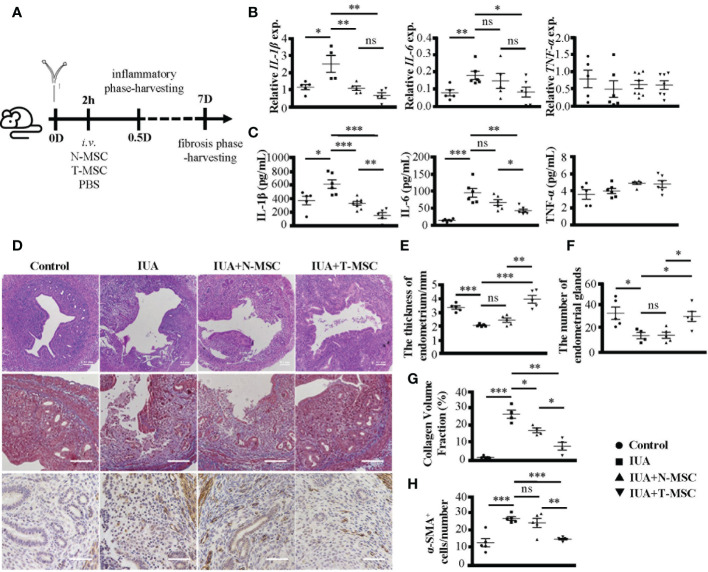
TNF-α pretreatment enhances the therapeutic efficacy of MSCs in IUA mice. **(A)** The schematic diagram of modeling process. MSCs were stimulated by TNF-α (10 ng/ml) for 24 h to prepare T-MSCs. Both N-MSCs and T-MSCs (1 × 10^6^ cells in 200 μl of PBS) were injected through the tail vein 2 h after the operation. **(B)** Relative expression of mRNAs of IL-1β, IL-6, and TNF-α in murine uterine tissues. **(C)** The expression levels of IL-1β, IL-6, and TNF-α in serum detected by ELISA. **(D)** (i) The top row: H&E staining of murine uterine tissues. **(ii)** The middle row: Masson’s trichrome staining of murine uterine tissues. (iii) The bottom row: α-SMA protein expression detected by immunohistochemistry. **(E)** The statistical figure of the endometrial thickness. **(F)** The statistical figure of gland numbers. **(G)** The statistical figure of collagen volume fraction. **(H)** The statistical figure of numbers of α-SMA^+^ cells. Bar = 0.1 mm. The measurement data are presented as the means ± SEM, n = 6; *p < 0.05, **p < 0.01, ***p < 0.001. TNF-α, tumor necrosis factor-α; MSCs, mesenchymal stem cells; IUA, intrauterine adhesion; T-MSCs, tumor necrosis factor-α-primed MSCs; N-MSCs, naïve MSCs; PBS, phosphate-buffered saline. ns, no significance.

Subsequently, we treated IUA mice with T-MSCs and compared their effect with N-MSCs (**Figure 3A**). In the early inflammatory phase of IUA, both N-MSCs and T-MSCs could reduce the expression levels of IL-1β and IL-6 in uterine tissues (**Figure 3B**) and serum (**Figure 3C**) but did not influence TNF-α. However, T-MSCs showed a strong anti-inflammatory ability. Similarly, in the late fibrosis period of IUA, T-MSCs could reverse uterine pathological changes better, including endometrial structure damage (**Figures 3D**, the top row, **E, F**), collagen deposition (**Figures 3D**, the middle row, in **Figure 3G**), and protein levels of α-SMA in uterine tissues (**Figure 3D**, the bottom row, in **Figure 3H**). These findings indicated that TNF-α pretreatment could effectively enhance the therapeutic activity of MSCs in IUA mice.

### 3.4 Tumor necrosis factor-α-primed mesenchymal stem cells promote macrophage polarization to M2 phenotype through exosomes

**Figure 4:**
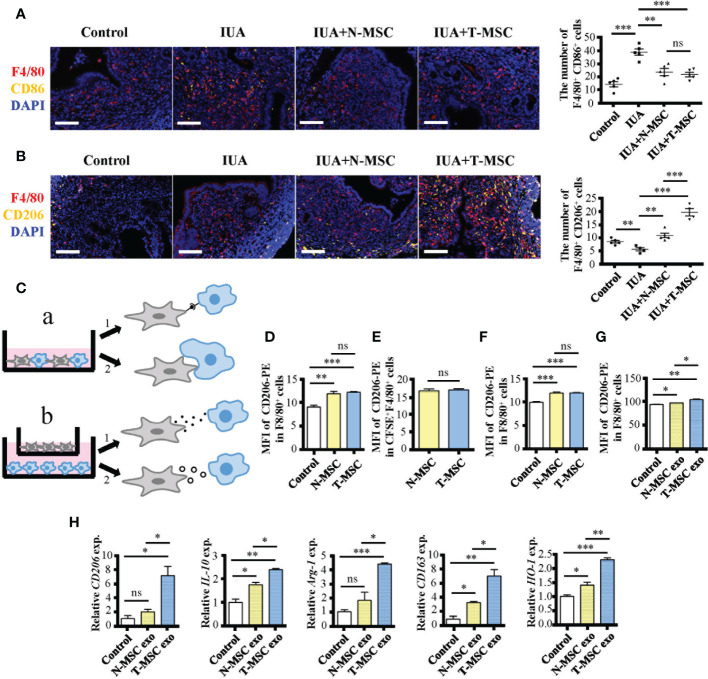
T-MSCs promote macrophage polarization to M2 phenotype through exosomes. **(A)** Immunofluorescence staining of F4/80 and CD86 proteins in murine uterine tissues after IUA modeling for 0.5 days. On the right is the statistical figure of numbers of F4/80^+^ CD86^+^ cells. **(B)** Immunofluorescence staining of F4/80 and CD206 proteins in murine uterine tissues after IUA modeling for 0.5 days. On the right is the statistical figure of numbers of F4/80^+^ CD206^+^ cells. **(C)** The schematic diagram of four ways for MSCs to play the immunosuppressive roles on macrophages including direct contact, efferocytosis, paracrine, and exosomes. **(D–G)** The CD206 expression level on F4/80^+^ macrophages was detected by flow cytometry assay. **(D)** MSCs were added into mouse peritoneal macrophage culture environment (1:10) directly for 24 h. **(E)** CFSE-marked MSCs were added into mouse peritoneal macrophage culture environment (1:10) directly for 24 h. The CFSE^+^ macrophages were considered macrophages that have engulfed MSCs. **(F)** MSCs were added into macrophage culture environment (1:10) with transwell for 24 h. **(G)** The MSC-derived exosomes (25 μg/ml) were added into mouse peritoneal macrophage culture environment for 24 h. **(H)** Relative expression of mRNAs of CD206, IL-10, Arg-1, CD163, and HO-1 in exosome-treated mouse peritoneal macrophages. Bar = 0.1 mm. The measurement data are presented as the means ± SEM, n = 3; *p < 0.05, **p < 0.01, ***p < 0.001. MSCs, mesenchymal stem cells; T-MSCs, tumor necrosis factor-α-primed MSCs; IUA, intrauterine adhesion; CFSE, carboxyfluorescein succinimidyl ester. ns, no significance.

IUA is a disease caused by damage or infection of the endometrium. As mentioned before, there was acute inflammation in the early stage of IUA. Moreover, the number of M1 macrophages (F4/80^+^ CD86^+^) increased while that of M2 macrophages (F4/80^+^ CD206^+^) decreased in IUA uterine tissues (**Figures 4A, B**). These phenomena were consistent with changes in the number of macrophages in the MDLI-IUA model (**Supplementary Figures 1K, L**). After treatment with MSCs, the number of M1 macrophages decreased in either the N- or T-MSC group with no significant difference (**Figure 4A**), and the number of M2 macrophages increased in the N- and T-MSC groups and more significantly in the T-MSC group (**Figure 4B**). These data suggested that T-MSCs might have a higher ability in promoting macrophage polarization to the M2 phenotype, which gave it a better therapeutic activity in IUA.

MSCs can play various immunosuppressive roles (35). To explore the key difference between T- and N-MSCs in immunosuppressive behaviors, we investigated several aspects such as direct contact (**Figures 4C** a1, **D**), efferocytosis (**Figures 4C** a2, **E**), paracrine (**Figures 4C** b1, **F**), and exosomes (**Figures 4C** b2, **G, H**). All experiments were applied with purified peritoneal macrophages *in vitro*. Among them, all MSC treatments could promote the expression of CD206 on macrophages, and there was no difference between the two treated groups in the first three cases (**Figures 4D–F**). However, T-MSC-derived exosomes showed a better ability to increase CD206 expression in macrophages than in N-MSC-derived exosomes (**Figure 4G**). Additionally, the mRNA expression of M2 markers such as CD206, IL-10, Arg-1, CD163, and HO-1 in macrophages was significantly upregulated in T-MSC-derived exosomes (**Figure 4H**). These results showed that the superiority of T-MSCs in promoting macrophage polarization to the M2 phenotype was mainly reflected in its exosomes.

### 3.5 Upregulated Galectin-1 in exosomes participates in immunosuppressive function of tumor necrosis factor-α-primed mesenchymal stem cells

**Figure 5:**
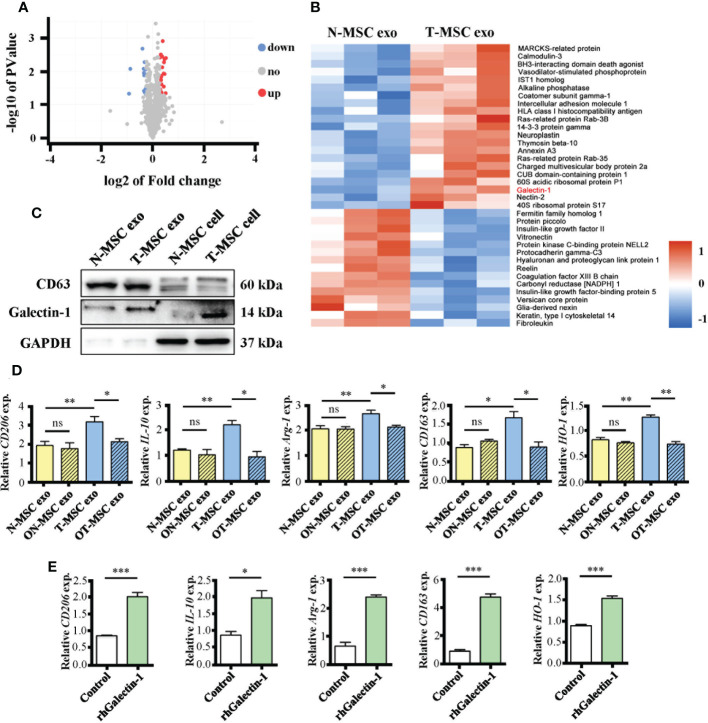
The upregulated Galectin-1 in exosomes participates in immunosuppressive function of T-MSCs. **(A, B)** Proteomic analysis of exosomes from T-MSC group and N-MSC group. **(A)** Volcano plots for the distribution of differentially expressed proteins. The blue part represents downregulation of expression, the red part represents upregulation, and the gray part represents no significance. n = 3. **(B)** Heatmap of 37 differentially expressed proteins between T-MSCs and N-MSCs. The blue part represents downregulation of expression. The red part represents upregulation. **(C)** The protein expression of CD63, Galectin-1 (Gal-1), and GAPDH were detected by Western blotting assay. **(D)** Relative expression of mRNAs of CD206, IL-10, Arg-1, CD163, and HO-1 in exosome-treated macrophages for 24 h. The ON-MSCs and OT-MSCs mean the N- or T-MSCs with OTX008 (60 μM) pretreatment for 1 h, followed by stimulation with or without TNF-α (10 ng/ml) for 24 h. **(H)** Relative expression of mRNAs of CD206, IL-10, Arg-1, CD163, and HO-1 in rhGalectin-1 protein (1 μg/ml)-treated mouse peritoneal macrophages for 24 h. The measurement data are presented as the means ± SEM, n = 3; *p < 0.05, **p < 0.01, ***p < 0.001. MSCs, mesenchymal stem cells; T-MSCs, tumor necrosis factor-α-primed MSCs; N-MSCs, naïve MSCs. ns, no significance.

To investigate T-MSC exosomes, which have a higher immunomodulatory capacity than N-MSC exosomes, we used mass spectrometric detection to analyze changes in their protein content. Without changing the particle size (**Supplementary Figures 3A, B**) or characteristic markers CD63 and CD9 (**Supplementary Figure 2C**), TNF-α pretreatment upregulated 21 molecules and downregulated 16 molecules in MSC-derived exosomes (**Figure 5A**). Cluster heatmap showed that Galectin-1 was significantly increased in T-MSC exosomes (**Figure 5B**), which was further verified *via* Western blotting (**Figure 5C**). In T-MSC exosomes, the expression of the exosome marker CD63 was consistent with that of N-MSC exosomes, while the expression of Galectin-1 was higher than that of the latter. Additionally, the intracellular Galectin-1 level of T-MSCs was higher than that of N-MSCs. Furthermore, Galectin-1 content in cell culture supernatant showed no difference (**Supplementary Figure 3D**). Considering previous results (**Figures 4C–H**), Galectin-1 was found to be important. OTX008 is a selective inhibitor of Galectin-1. In promoting macrophage polarization to the M2 phenotype, OTX008 pretreatment could reverse the effect of TNF-α (**Figure 5D**). Additionally, stimulation of rhGalectin-1 protein upregulated the expression of M2 phenotype-related genes in macrophages (**Figure 5E**). These findings suggested that Galectin-1 was upregulated in T-MSC exosomes and that it participates in the immunosuppressive function of T-MSCs.

### 3.6 Galectin-1 promotes polarization of macrophages to M2 mainly through the Jak-STAT signaling pathway

**Figure 6:**
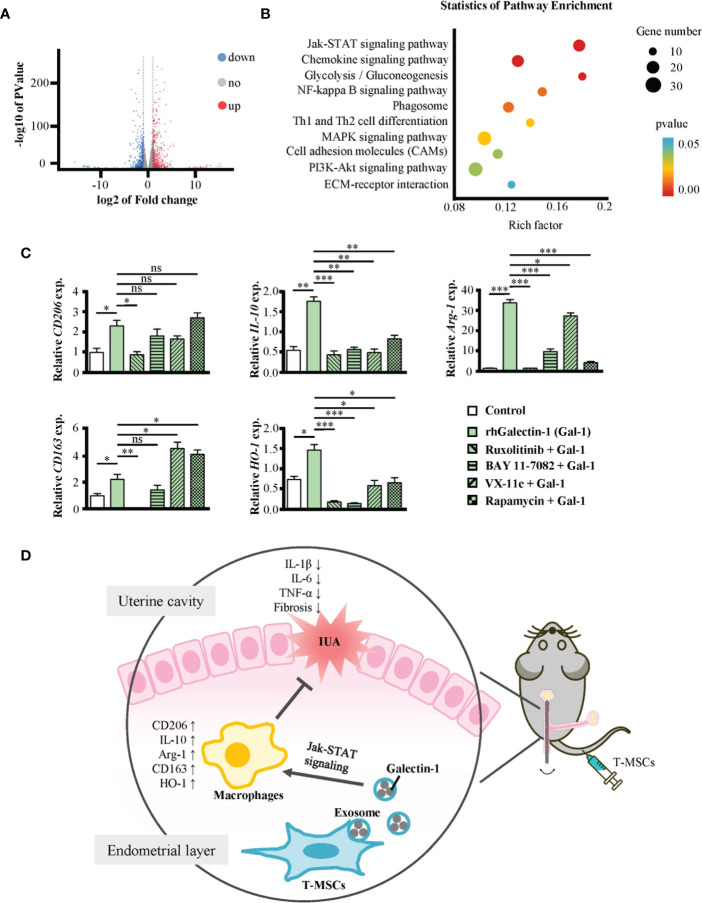
Galectin-1 promoted macrophages to M2 mainly through Jak-STAT signaling pathway. **(A, B)** RNA-seq analysis of mouse peritoneal macrophages treated with or without rhGalectin-1 protein (1 μg/ml) for 24 h. **(A)** Volcano plots for the distribution of differentially expressed genes (Galectin-1 group/control group). The blue part represents downregulation of expression, the red part represents upregulation, and the gray part represents no significance. n = 3. **(B)** The KEGG pathways analysis of differentially expressed genes between Galectin-1 group and control group. The red part indicates the differential gene is extremely significantly enriched KEGG classification (p < 0.01); the blue part indicates the differential gene is significantly enriched KEGG classification (p < 0.05). The size of the circle represents the number of genes. **(C)** Mouse peritoneal macrophages were pretreated with or without ruxolitinib (1 μmol/L), BAY 11-7082 (1 μmol/L), VX-11e (100 nmol/L), and rapamycin (10 μmol/L) for 1 h, followed by stimulation with rhGalectin-1 protein (1 μg/ml) for 24 h. The mRNA expression levels of CD206, IL-10, Arg-1, CD163, and HO-1 in mouse peritoneal macrophages were determined by qRT-PCR. **(D)** Full-text summary diagram. The measurement data are presented as the means ± SEM, n = 3; *p < 0.05, **p < 0.01, ***p < 0.001. KEGG, Kyoto Encyclopedia of Genes and Genomes. ns, no significance.

To investigate the signaling pathway through which Galectin-1 promoted the polarization of macrophages toward the M2 phenotype, we obtained their gene expression profile *via* RNA sequencing technology. The results showed 1,239 differentially expressed genes, among which 650 genes were upregulated and 589 genes were downregulated in Galectin-1-treated macrophages (**Figure 6A**). We analyzed the differential expression genes, and the results are shown on a heatmap (**Supplementary Figure 5A**). Subsequently, these candidates underwent secondary screening using the Kyoto Encyclopedia of Genes and Genomes (KEGG) pathway enrichment to determine the core signaling pathways (**Figure 6B**). According to the results, Galectin-1 treatment significantly influenced many aspects of macrophages, including the Jak-STAT signaling pathway, NF-κB signaling pathway, phagosome, Th1 and Th2 cell differentiation, MAPK signaling pathway, and PI3K-Akt signaling pathway.

To further find out the pathway through which Galectin-1 promotes M2 polarization of macrophages, we pretreated macrophages with ruxolitinib (inhibitor of Jak1/2), BAY 11-7082 (inhibitor of NF-κB), VX-11e (inhibitor of ERK1/2), and rapamycin (inhibitor of mTOR) for 1 h and then treated them with rhGalectin-1 for 24 h (**Figure 6C**). All these inhibitors inhibited the mRNA expression of M2-type markers in macrophages to varying degrees. Among them, ruxolitinib was the most significant. In summary, Galectin-1 promoted the polarization of macrophages to M2 through several signaling pathways and mainly through the Jak-STAT signaling pathway.

## 4 Discussion

IUA is caused by severe damage or infection of the endometrium, its normal structural cell proliferation, differentiation disorders, fibrous tissue replacement, and the formation of adhesions and scars (5). The etiology of IUA is relatively clear, but its pathophysiology is complex. Therefore, establishing an accurate animal model is essential for studying the mechanism, prevention, and treatment of IUA. The existing IUA modeling methods include mechanical damage with various homemade curettes and scalpels, electrical burns, chemical damage from alcohol or LPS, and a combination of several approaches (32, 36, 37). Among them, the most used is the MDLI-IUA model (38). However, in all these methods, there is a need to open the abdominal cavity to expose the uterus, which causes the following problems: i) long operation time increases the chances of animals dying during surgery; ii) bacterial infections can occur, causing other complications such as peritonitis; iii) abdominal wounds can bring a series of immune responses, which might interfere with the immunological study; iv) the modeling process is complex and time-consuming. Additionally, the most common IUA model using mechanical damage plus LPS infection also has two shortcomings: i) scratching the uterus with a hand is difficult due to the inability of maintaining consistent strength, affecting the repeatability of modeling; ii) artificial addition of LPS does not simulate the actual clinical situation.

In this study, we proposed an improved method to build a murine IUA model called the electric tool-scratching IUA model. It had the following advantages: i) it abandons the process of opening the abdominal cavity, which is less painful and more consistent with animal welfare requirements; ii) it avoids unnecessary damage other than uterine cavity injury; iii) it not only can avoid the death of model animals but also can reduce interference factors in the study; iv) it has uniform scratching strength and is easy to standardize while showing the same effect as the traditional model; v) it is a high-speed and time-saving operation. The electric tool-scratching IUA model can achieve nearly identical effects as the mechanical damage–LPS infection model, including increased inflammation, damaged endometrial morphology, and fibrosis. Based on the above, our electric tool-scratching IUA model can efficiently improve the development of the murine IUA model and contribute to the study of its pathological mechanism.

However, this approach has some drawbacks. On the one hand, as there was no LPS intervention, the IUA model showed milder symptoms, and the levels of inflammatory factors such as IL-1β, IL-6, and TNF-α and the degree of endometrial fibrosis were lower than those in the MDLI-IUA model. Therefore, a new model is more suitable for studying mild clinical cases. On the other hand, due to the restriction of no laparotomy, we could only damage one side of the uterine horn with a tool. The other side was used as a blank control to evaluate pathological changes.

Many studies have reported that MSC therapy promotes the reconstruction of damaged endometrium (39, 40). We previously found that MSC-combined collagen scaffolds can significantly reduce endometrium fibrosis and improve the pregnancy rate in a rat model of intrauterine adhesion (10). Excitingly, autologous stem cells loaded onto collagen scaffolds successfully alleviated endometrium fibrosis and regeneration in IUA patients (11). However, it is puzzling that a considerable number of patients still do not respond well to MSC-collagen scaffolds. Therefore, how to further improve the ability of MSCs to repair the endometrium has become our current focus.

MSCs are a group of heterogeneous cells. With changes in the microenvironment, MSCs can secrete a variety of substances to regulate immunity, reduce inflammation, and promote tissue healing (41). We previously found that miRNA treatment of MSCs can affect their proliferation and function (13, 14); IL-1β pretreatment can enhance the migration ability and immunosuppressive effect of MSCs (15), and exosomal miR-146a contributes to the enhanced therapeutic efficacy of IL-1β-primed MSCs against sepsis (12). These studies suggested that the anti-inflammatory and repair functions of MSCs depend on the microenvironment and its activation state. It was predicted that the immunosuppressive function of MSCs could be increased by TNF-α released by the transplant host (17). To improve the efficacy of MSC-based therapies for IUA, we pretreated MSCs using TNF-α and named them T-MSCs. Since the model animal was a mouse, mouse TNF-α was selected. In this study, TNF-α did not affect the stem cell phenotype, cell viability, and cell apoptosis of MSCs. T-MSCs showed a higher ability to promote macrophage polarization to M2 phenotype and better therapeutic efficacy on IUA. Moreover, pretreated MSCs using human TNF-α could also enhance their effectiveness in both treatment of IUA and the promotion of macrophage M2 polarization without changing the phenotype of MSCs (**Supplementary Figure 4**). These results together demonstrated the enhancing effect of TNF-α on the functions of MSCs. Additionally, the expression of TRAF-1 was upregulated in T-MSCs. Our previous study showed that TRAF1 expression in MSCs is critical for the promotion of macrophage M2 polarization and the alleviation of sepsis (42). Therefore, the excellent efficacy of T-MSCs may be related to this.

There are several potential mechanisms for MSCs to exert immunomodulatory effects, for example, dying-cell clearance *via* phagocytosis (efferocytosis) and paracrine signaling including exosomes (26) and soluble molecules (35). To investigate the reason why T-MSCs are superior to naïve MSCs in promoting M2-type polarization of macrophages, we performed *in vitro* co-culture experiments. By co-culturing MSCs with mouse primary peritoneal macrophages in different ways, we found that the superiority of T-MSCs is only related to exosomes. Proteomic results showed that the expression of many proteins, including Galectin-1 (Gal-1), MARCKS-related protein, calmodulin-3, intercellular adhesion molecule 1 (ICAM-1), Ras-related protein Rab-3B, neuroplastin, thymosin beta-10, charged multivesicular body protein 2a, and Nectin-2, increased in T-MSC exosomes. Most of these proteins are poorly studied. Among them, ICAM-1 is a cell surface glycoprotein and an adhesion receptor, which is usually expressed on the cell surface and plays an important role (43). Neuroplastin is mainly involved in neuropsychiatric diseases (44). However, Galectin is the main regulatory molecule for MSCs to exert immunosuppressive function, and MSCs can express different types of galectins (45). Studies have also shown that Gal-1 is a local immunomodulatory factor in MSCs (46) and that Gal-1 in BMSCs is related to their intra-articular immunomodulatory properties (45). Additionally, several reports have suggested that Gal-1 has anti-inflammatory properties on macrophages (29–31). The expression difference of Galectin-1 was not the most significant, but its immunological function was the most prominent. Hence, we considered Gal-1 as the most important molecule in T-MSC exosomes, and the following study was performed on it.

After inhibiting the expression of Galectin-1 with OTX008, the superior immunosuppressive function of T-MSCs disappeared. These results demonstrated that the strong immunomodulatory capacity of T-MSC could be attributed to Galectin-1 in exosomes. However, we did not inject T-MSC exosomes to treat IUA mice directly, which should be systematically investigated in further studies. Moreover, the treatment of macrophages with recombinant human Galectin-1 enabled their M2 polarization. RNA-seq data and inhibitor experiments showed that many signaling pathways were involved in the effect of Gal-1 on macrophages, including the Jak-STAT signaling pathway, NF-κB signaling pathway, phagosome, MAPK signaling pathway, and PI3K-Akt signaling pathway. Among them, the Jak-STAT signaling pathway was the most important signaling pathway.

In addition to changes in signaling pathways, RNA-seq data demonstrated the effect of Galectin-1 on gene expression patterns in macrophages. Gene Ontology (GO) enrichment analysis of differential expression genes revealed that they were mainly related to the cellular response to lipopolysaccharide (39 genes), immune system progress (68 genes), and response to the virus (26 genes) (**Supplementary Figures 5B, C**). After re-analyzing the differentially expressed genes of these three gene sets, we found that their intersection was LCN2 and Src (**Supplementary Figure 5D**). Between them, Lcn2 had a higher expression level. The Lcn2 gene encodes a lipocalin-2 protein (LCN2), which has multiple immune effects. LCN2 has been reported to promote wound repair, and an LCN2-blocking antibody significantly inhibits skin wound repair (35). Several studies have shown that LCN-2 has protective effects on infections and inflammatory bowel disease (47). IL-10 and LCN2 double-knockout mice have more severe colitis and a higher expression of inflammatory factors (48). Knockdown of Lcn2 promotes M1-type polarization and upregulation of inflammatory factors such as IL-1β, IL-6, and TNF-α in macrophages after LPS stimulation (49). These studies showed that LCN2 might be an essential effector molecule in the anti-inflammation and repair progress of macrophages. Its specific role in IUA treatment still needs to be further studied.

In summary, our studies proposed a novel method to build a murine IUA model with the advantages of being minimally invasive, efficient, and controllable. Moreover, we revealed that TNF-α pretreatment enhanced the therapeutic efficacy of MSCs on IUA. Mechanistically, Galectin-1 in exosomes of T-MSCs promoted endometrial macrophage polarization to M2 phenotype mainly through the Jak-STAT signaling pathway (**Figure 6D**). These findings may contribute to the study of the pathological mechanism and treatment strategies of IUA.

## Data availability statement

The original contributions presented in the study are included in the article/**Supplementary Material**. Further inquiries can be directed to the corresponding authors.

## Ethics statement

The animal study was reviewed and approved by Model Animal Research Center of Nanjing University.

## Author contributions

JL performed and analyzed all the experiments and wrote the manuscript. YP and JW supported the materials. JY and QJ participated in the animal experiments. YH, HD and JL conceived and designed the project. YH and JL co-designed experiments and co-wrote the manuscript. All authors contributed to the article and approved the submitted version.
