# Supplementary material for: Tumor necrosis factor-α-primed mesenchymal stem cell-derived exosomes promote M2 macrophage polarization via Galectin-1 and modify intrauterine adhesion on a novel murine model
Source: Front Immunol. 2022 Dec 16;13:945234. doi: 10.3389/fimmu.2022.945234 (PMC9800892; doi:10.3389/fimmu.2022.945234)
Supplement: Supplementary file 1 [file DataSheet_1.zip › Supplementary Material/Supplementary_Material.docx]

Supplementary Material

# Supplementary Figures and Tables

## Supplementary Figures

**Supplementary Figure 1. Pathological features of the MDLI-IUA mice model. (A)** The schematic diagram of modelling process. IUA mice model was established by mechanical damage-LPS infection way mentioned before. The early inflammatory phase and late fibrosis period respectively are 0.5 days and 7 days after modeling. **(B)** H&E staining of murine uterine tissue. **(C)** The statistical figure of the endometrial thickness. **(D)** The statistical figure of gland numbers. **(E)** Relative expression of mRNAs of IL-1β, IL-6 and TNF-α in murine uterine tissue detected by qRT-PCR assay. **(F)** The level of IL-1β, IL-6 and TNF-α in serum detected by ELISA assay. **(G)** Masson’s trichrome staining of murine uterine tissue, and statistical figure of collagen volume fraction. **(H)** The α-SMA protein expression detected by immunohistochemistry, and statistical figure of numbers of α-SMA^+^ cells. **(I)** The PCNA protein expression detected by immunohistochemistry. **(J)** The CK19 protein expression detected by immunohistochemistry. **(K)** Immunofluorescence staining of F4/80 and CD86 proteins in murine uterine tissue after IUA modeling for 0.5 days. **(L)** Immunofluorescence staining of F4/80 and CD206 proteins in murine uterine tissue after IUA modeling for 0.5 days. Bar=0.1 mm. The measurement data are presented as the means ± SEM, n = 6; *P< 0.05, **P< 0.01, ***P < 0.001.

**Supplementary Figure 2. The effect of TNF-α treatment on MSCs. (A)** Flow cytometry assay results showed the expression of CD34, CD45, HLA-DR, CD73, CD90 and CD105 of MSCs treated with TNF-α (10ng/mL) for 24h. **(B)** The cell viability detected by CCK-8 assay on MSCs treated with various concentrations of TNF-α for 24h. **(C)** Flow cytometry assay results showed the apoptosis of MSCs treated with or without of TNF-α (10ng/mL) for 24h. **(D)** Relative expression of mRNAs of TRAF-1 in MSCs treated with or without TNF-α (10ng/mL) for 24h. **(E)** Immunofluorescence staining of TRAF-1 protein on MSCs treated with or without TNF-α (10ng/mL) for 24h. Bar=0.1 mm. The measurement data are presented as the means ± SEM, n = 3; *P< 0.05, **P< 0.01, ***P < 0.001.

**Supplementary Figure 3.** **Characterization of Exosomes.** (A) The particle sizes of N- and T-MSC exosomes were detected by Nanoflowmeter. **(B)** Transmission electron micrograph of N- and T-MSC exosomes. Bar=0.1 mm. **(C)** The characteristic markers CD63 and CD9 of N- and T-MSC exosomes were detected by Nanoflowmeter. **(D)** The expression of Galectin-1 in MSC culture supernatant were detected by ELISA. The measurement data are presented as the means ± SEM, n = 3; *P< 0.05, **P< 0.01, ***P < 0.001.

**Supplementary Figure 4. The effect of human TNF-α pretreated MSCs (hT-MSC) on IUA mice. (A)** Flow cytometry assay results showed the expression of CD34, CD45, HLA-DR, CD73, CD90 and CD105 of MSCs treated with human TNF-α (10ng/mL) for 24h. **(B)** Relative expression of mRNAs of IL-1β, IL-6 and TNF-α in murine uterine tissue detected by qRT-PCR assay. n = 4 **(C)** The level of IL-1β, IL-6 and TNF-α in serum detected by ELISA assay. n = 4 **(D)** Relative expression of mRNAs of CD206, IL-10, Arg-1, CD163 and HO-1 in hT-MSC exosomes treated mouse peritoneal macrophages. n = 3. The measurement data are presented as the means ± SEM; *P< 0.05, **P< 0.01, ***P < 0.001.

**Supplementary Figure 5. Gene expression pattern of Galectin-1 treated macrophages. (A)** Heatmap of a part of differentially expressed genes between Galectin-1 group and Control group (n = 3). The blue part represents down regulation of expression. The red part represents upregulation. **(**B**)** The GO enrichment analysis of differentially expressed genes between Galectin-1 group and Control group. The red part indicates the differential gene is extremely significantly enriched GO classification (P <0.01); the green part indicates the differential gene is significantly enriched GO classification (P <0.05); The blue part is enriched in GO classification with no significant differences. The size of the circle represents the number of genes. **(C)** Gene intersections of gene sets associated with cellular response to lipopolysaccharide, immune system progress and response to virus. **(D)** The bar graph displays the two genes commonly expressed in them.

**Supplementary Table 1** Primer sequences used in RT-PCR

| Gene | primer sequence |
| --- | --- |
| Mouse IL-1β | Forward: TTCAGGCAGGCAGTATCACTC  Reverse: GAAGGTCCACGGGAAAGACAC |
| Mouse IL-6 | Forward: TAGTCCTTCCTACCCCAATTTCC  Reverse: TTGGTCCTTAGCCACTCCTTC |
| Mouse TNF-α | Forward: CCCTCACACTCAGATCATCTTCT  Reverse: GCTACGACGTGGGCTACAG |
| Mouse CD206 | Forward: GCTTCCGTCACCCTGTATGC  Reverse: TCATCCGTGGTTCCATAGACC |
| Mouse IL-10 | Forward: GCTCTTACTGACTGGCATGAG  Reverse: CGCAGCTCTAGGAGCATGTG |
| Mouse Arg-1 | Forward: CTCCAAGCCAAAGTCCTTAGAG  Reverse: AGGAGCTGTCATTAGGGACATC |
| Mouse CD163 | Forward: ATGGGTGGACACAGAATGGTT  Reverse: CAGGAGCGTTAGTGACAGCAG |
| Mouse HO-1 | Forward: AAGCCGAGAATGCTGAGTTCA  Reverse: GCCGTGTAGATATGGTACAAGGA |
| Mouse β-actin | Forward: AGGTGACAGCATTGCTTCTG  Reverse: GGGAGACCAAAGCCTTCATA |
| Human TRAF-1 | Forward: TGAGAGGGGAGTATGATGCG  Reverse: GACGCTGAGCTTAGGTCAGG |
| Human GAPDH | Forward: GGAGCGAGATCCCTCCAAAAT  Reverse: GGCTGTTGTCATACTTCTCATGG |
