# Supplementary figures and images for: Tumor necrosis factor-α-primed mesenchymal stem cell-derived exosomes promote M2 macrophage polarization via Galectin-1 and modify intrauterine adhesion on a novel murine model
Source: Front Immunol. 2022 Dec 16;13:945234. doi: 10.3389/fimmu.2022.945234 (PMC9800892; doi:10.3389/fimmu.2022.945234)

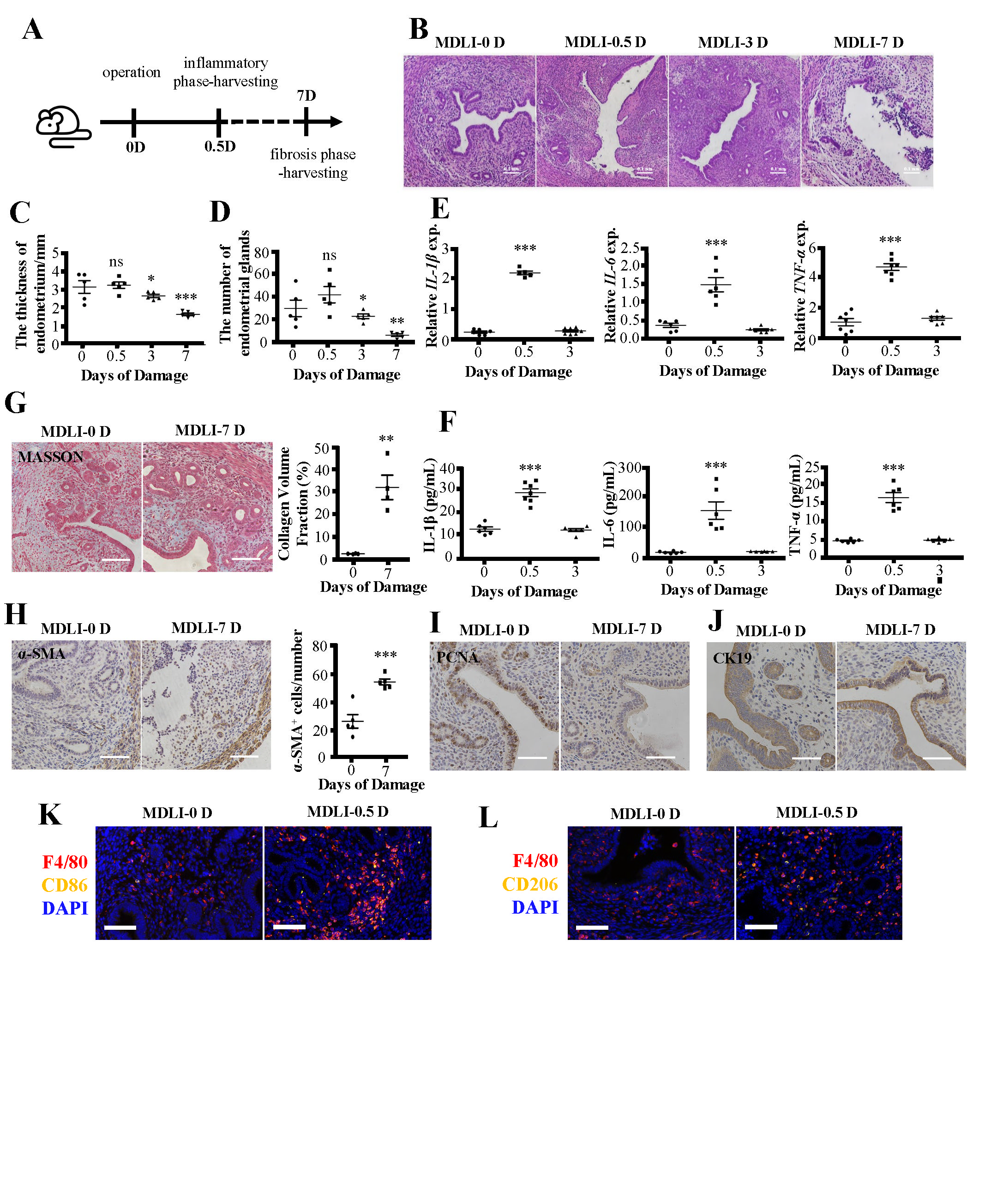

Supplement: Supplementary file 1 [file DataSheet_1.zip › Supplementary Material/Supplementary Figures/SFig.1.tif]

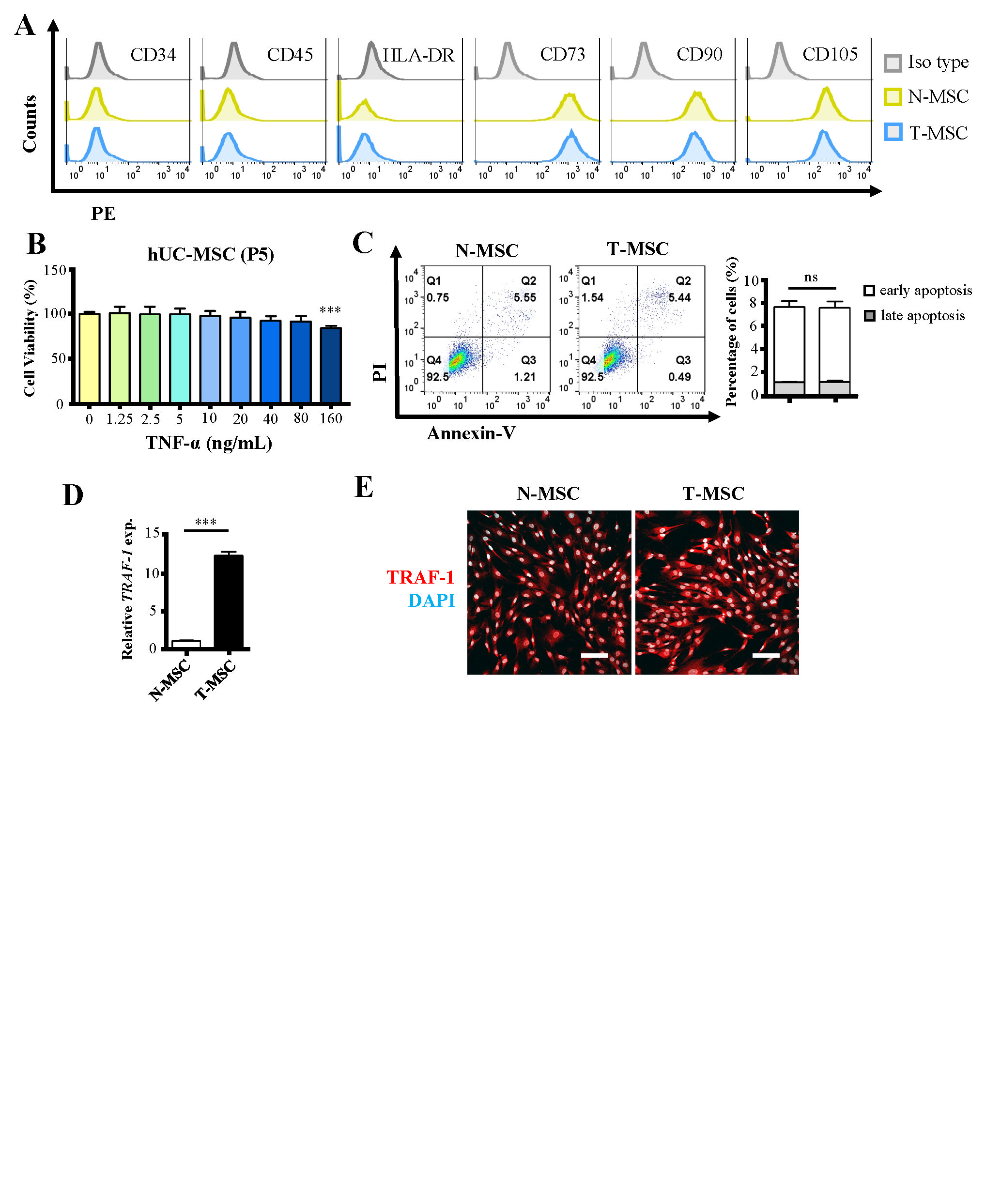

Supplement: Supplementary file 1 [file DataSheet_1.zip › Supplementary Material/Supplementary Figures/SFig.2.tif]

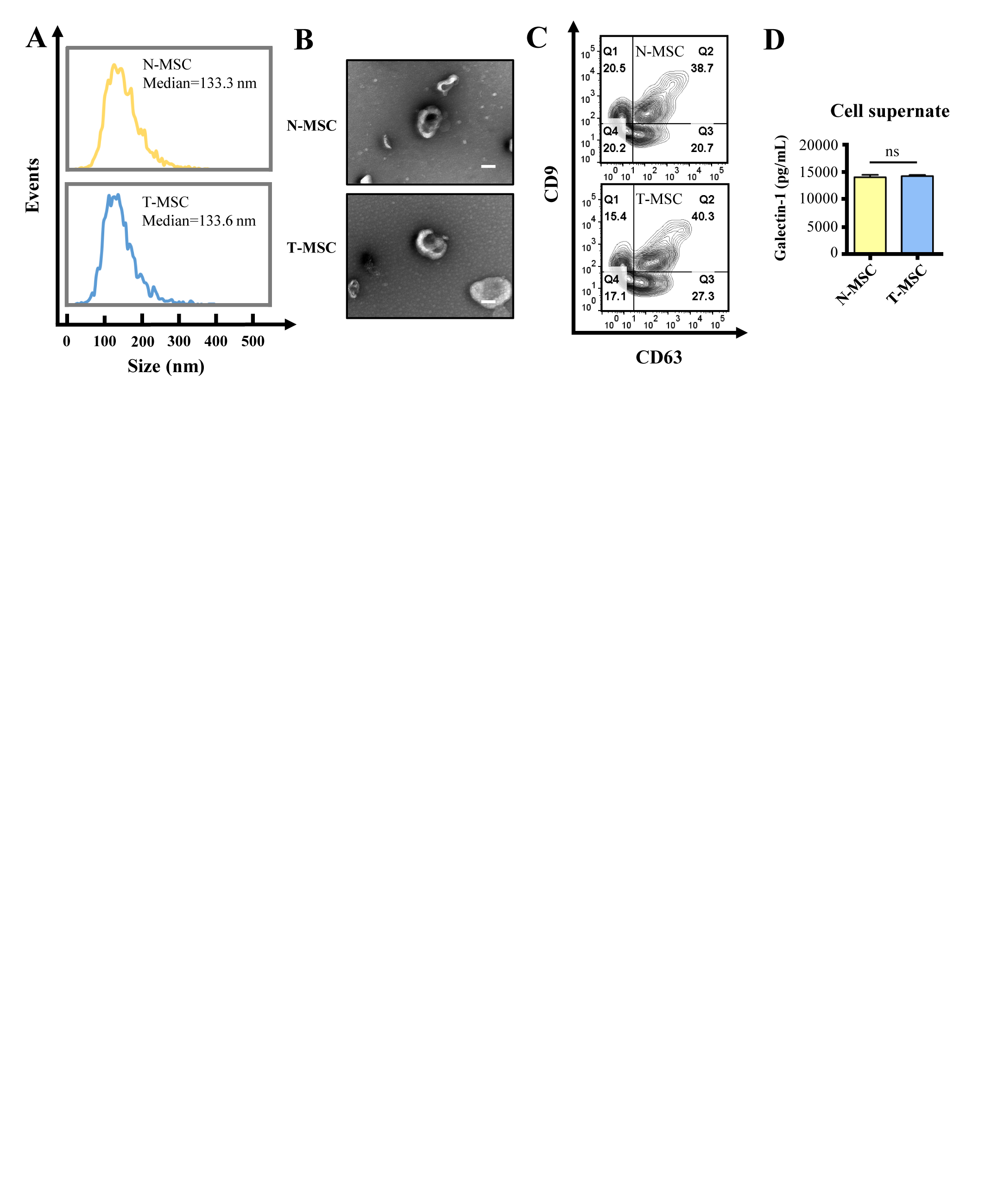

Supplement: Supplementary file 1 [file DataSheet_1.zip › Supplementary Material/Supplementary Figures/SFig.3.tif]

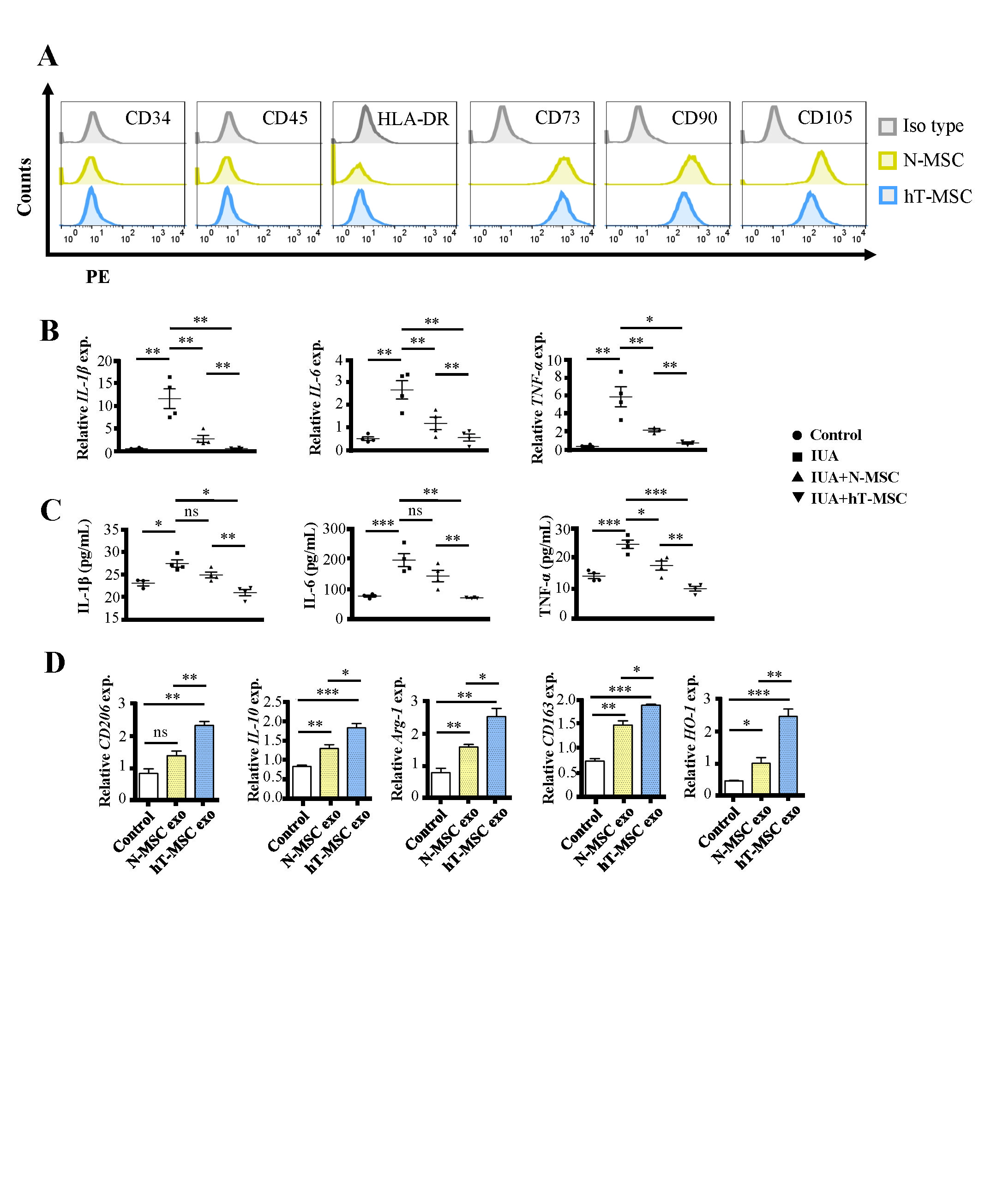

Supplement: Supplementary file 1 [file DataSheet_1.zip › Supplementary Material/Supplementary Figures/SFig.4.tif]

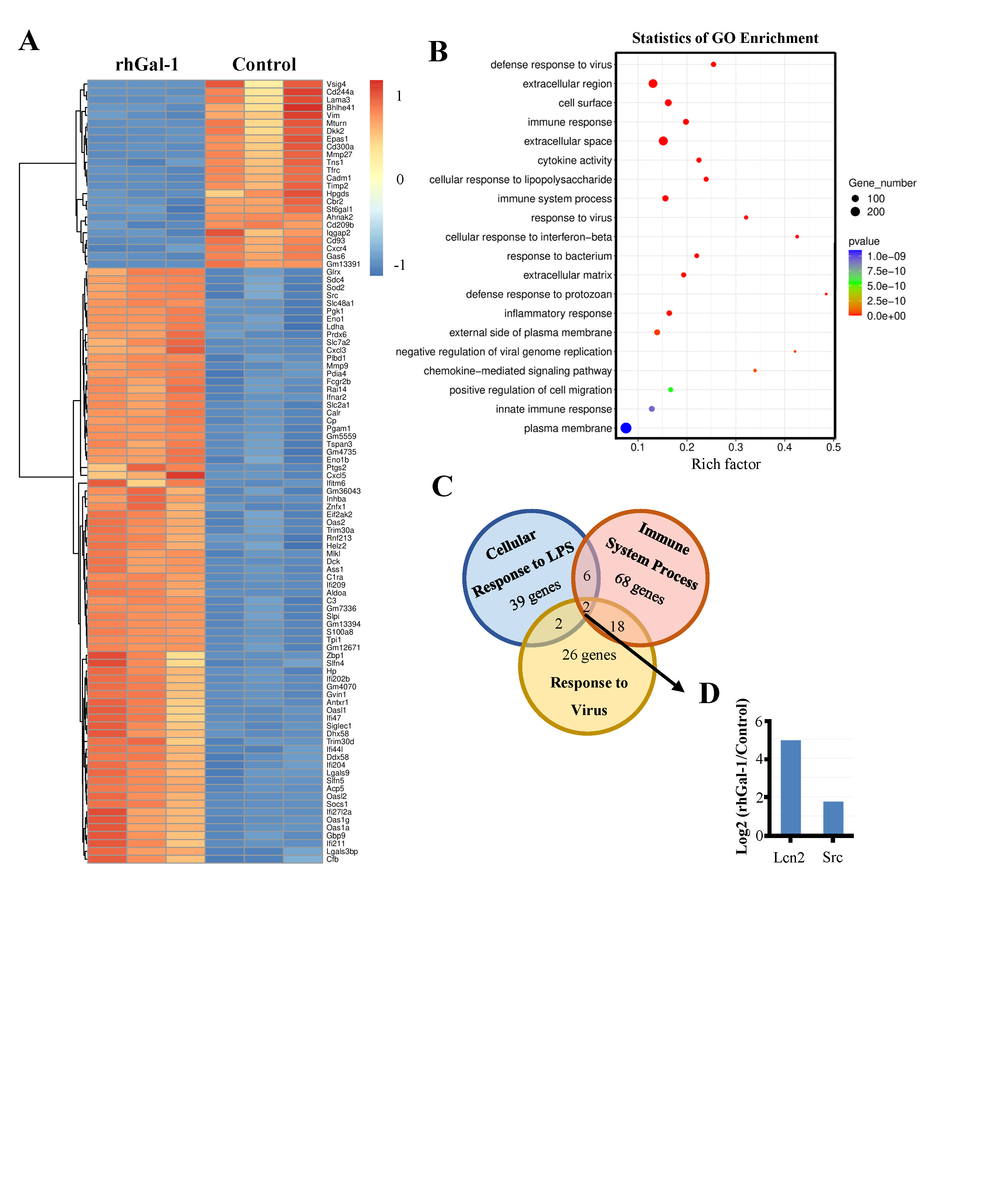

Supplement: Supplementary file 1 [file DataSheet_1.zip › Supplementary Material/Supplementary Figures/SFig.5.tif]
